# Hidden lesions: a case of burnt remains

**DOI:** 10.1093/fsr/owad019

**Published:** 2023-06-23

**Authors:** Negahnaz Moghaddam, Lorenzo Campana, Claudine Abegg, Raquel Vilarino, Christelle Voland, Fabrice Dedouit, Pia Genet, Tony Fracasso

**Affiliations:** Unit of Forensic Imaging and Anthropology, University Centre of Legal Medicine Lausanne-Geneva, Lausanne University Hospital and University of Lausanne, Switzerland; Swiss Human Institute of Forensic Taphonomy, University Centre of Legal Medicine Lausanne-Geneva, Lausanne University Hospital and University of Lausanne, Switzerland; Unit of Forensic Imaging and Anthropology, University Centre of Legal Medicine Lausanne-Geneva, Lausanne University Hospital and University of Lausanne, Switzerland; Unit of Forensic Imaging and Anthropology, University Centre of Legal Medicine Lausanne-Geneva, Lausanne University Hospital and University of Lausanne, Switzerland; Unit of Forensic Medicine, University Centre of Legal Medicine Lausanne-Geneva, Lausanne University Hospital and University of Lausanne, Switzerland; Unit of Forensic Medicine, University Centre of Legal Medicine Lausanne-Geneva, Lausanne University Hospital and University of Lausanne, Switzerland; Unit of Forensic Imaging and Anthropology, University Centre of Legal Medicine Lausanne-Geneva, Lausanne University Hospital and University of Lausanne, Switzerland; Unit of Forensic Imaging and Anthropology, University Centre of Legal Medicine Lausanne-Geneva, Geneva University Hospital and University of Geneva, Switzerland; Department of Forensic Pathology, Centre Hospitalier Universitaire de Toulouse, Hôpital Rangeuil, France; Department of Radiology, Centre Hospitalier de Toulouse, Hôpital Rangeuil, France; Unit of Forensic Imaging and Anthropology, University Centre of Legal Medicine Lausanne-Geneva, Lausanne University Hospital and University of Lausanne, Switzerland; Unit of Forensic Medicine, University Centre of Legal Medicine Lausanne-Geneva, Geneva University Hospital and University of Geneva, Switzerland; Unit of Forensic Medicine, University Centre of Legal Medicine Lausanne-Geneva, Lausanne University Hospital and University of Lausanne, Switzerland; Unit of Forensic Medicine, University Centre of Legal Medicine Lausanne-Geneva, Geneva University Hospital and University of Geneva, Switzerland

**Keywords:** burnt remains, forensic anthropology, forensic imaging, forensic pathology, trauma, fracture

## Abstract

One of the many challenging cases that forensic pathologists, anthropologists, and forensic imaging experts have to face are burnt human remains. Perpetrators frequently attempt to hide/destroy evidence and make the body unidentifiable by exposing it to fire. We present a case of a partially burnt body found in an apartment after an explosion. First, multidetector computed tomography (MDCT) images and the following autopsy revealed several lesions on the cranium. Forensic anthropologists were involved in order to specify the aetiology of the lesions observed on the cranium. Through an interdisciplinary approach bringing together MDCT scans, 3D surface scans, and anthropological analysis, it was possible to answer the questions raised during the autopsy. Analyses demonstrated that there were signs of blunt force trauma on the cranium vault that the perpetrator likely attempted to hide by exposing the body to fire. This case demonstrates the importance of close collaboration between forensic anthropologists, imaging experts, and forensic pathologists. This multidisciplinary approach allows for a better, more complete reconstitution of forensic cases.

**Key points:**

- The analyses of burnt human remains are one of the many challenging tasks that forensic pathologists and anthropologists have to face.
- We present an occurrence of a partially burnt body after an explosion and forensic anthropologists were asked whether the nature of the lesions observed on the cranium could be further specified.
- Anthropological analyses of the skull were consistent with the radiological and autopsy report. It was possible to reconstruct the various lesions on the dry bone.
- The case demonstrates the importance of an interdisciplinary approach and the close collaboration between forensic anthropologists, imaging experts, and forensic pathologists.

## Introduction

As described by Symes et al. [1], fire is a destructive force that can damage or even destroy important evidence. Exposing a dead body to fire is frequently attempted by perpetrators in order to make the body difficult to be recognized and/or to destroy evidences [1]. Indeed, the high temperature may cause important alterations to human tissue, which can lead to obstacles and difficulties during forensic investigations. Modifications to the bone may differ depending on the location, the temperature, and the duration of time during which the body was exposed to heat [2]. These alterations may also change the characteristics of various kinds of lesions, such as fractures.

Nevertheless, usually the temperature rarely destroys a body [1, 3] and heat-induced (HI) alterations may create distinctive patterns that may provide significant information on the circumstances of death. Pope and Smith [4] showed in their research the possibility of identifying pre-existing trauma in burnt cranial bones. However, a detailed research and correct interpretation of HI modifications is crucial for the complete understanding of the sequence of events. Therefore, extensive research has been conducted on the question of how to analyse, detect, and interpret lesions on burnt remains [4, 5, 6–10].

Forensic anthropologists possess detailed knowledge of the human skeleton and its variations. Specialists in forensic anthropology have experience on how various factors may influence human bones. When dealing with dead bodies that are no longer recognizable, such as skeletonized, highly fragmented, or particularly burnt remains, forensic anthropologists play an important role within the investigation process [11].

Multidetector computed tomography (MDCT) is of great importance when it comes to analysing a burnt body. It has therefore increasingly been used within fire deaths, as it allows pathologists to have a better overview of the lesion panel (fractures, organ lesions, etc.) and to detect foreign bodies [12–15]. Complementarily, 3D surface scans assist in getting higher resolution surfaces through the 3D acquisition of the lesion panel and offering an opportunity to create true to scale object-lesion shape comparisons within a virtual environment. Various studies have elaborated on the potential of MDCT and 3D surface scans in forensic anthropology in the past decades, and the discipline of virtual anthropology in particular has become an important part of forensic anthropology [16, 17–20].

We present here an occurrence of a partially burnt body in an apartment after an explosion. The close collaboration between forensic anthropologists, imaging experts, and forensic pathologists allowed a more complete understanding of the cases.

## Case report

Police and firefighters were called after an explosion in a family apartment. The cause of the explosion was first unknown. A body was found dead on the ground in the kitchen area and an alleged friend of the victim was uninjured waiting when the firefighter and the police arrived. The pathologists were subsequently called to the scene and proceeded with the first external examination. They discovered the charred body of an adult male lying on the ground with his elbows and wrists bent. Three openings caused by the fire were noted on the body, one of which contained the heart and the other two containing the digestive track.

The body was transferred to the medico-legal facility for autopsy and additional examinations of the cause and circumstances of death, upon request by the prosecutor. Several DNA (samples taken on the body and from blood spots on objects) and forensic odontological analyses were performed. Furthermore, an MDCT-scan of the body was performed before autopsy, and samples were taken for toxicological and histological analyses. MDCT images and the following autopsy revealed several lesions on the skull, including a potential blunt force trauma (BFT) to the left side of the skull. Based on the radiological examination and the autopsy, the case was treated as a suspected homicide.

After consultation with the responsible law enforcement, the police again interrogated the victims’ friend. The police found a hammer with blood spots and an empty bottle smelling of petrol in a bag belonging to the friend. He testified he had hit the victim several times on the head using a hammer. He dragged the unconscious body to the kitchen and set fire to the kitchen area, which eventually led to the explosion. The responsible pathologists got information about the confession the day after the autopsy. However, the initial auditions of the perpetrator were confused and unclear as to the exact sequence of actions executed. The decision was made by the prosecutor to request additional forensic specialist’s participation.

Forensic anthropologists were asked whether the nature of the lesions observed on the cranium could be further specified and to differentiate the lesions caused by the fire and possible BFT, which could have been related to the cause of death. Specialists in 3D reconstructions worked together with the anthropologists in order to establish 3D reconstructions of the lesions and to analyse whether the suspected hammer could be excluded as a weapon. Furthermore, the 3D images produced were used in order to assist the anthropologists in understanding whether multiple impacts were possible. The aim of the analysis was to answer the questions raised during the autopsy through an interdisciplinary approach bringing together MDCT- and 3D surface scans, and anthropological analysis.

## Material and methods

### Computed tomography of the body

All corpses are routinely MDCT scanned prior to autopsy at the Lausanne-Geneva University Centre for Forensic Medicine in Switzerland. An initial immediate radiological examination is performed prior to autopsy to detect foreign bodies as well as lesions and other noticeable features.

An MDCT Lightspeed VCT 64 (GE Healthcare, Milwaukee, WI, USA) was used for the MDCT scans. The images were analysed by a radiologist with experience in forensic medicine and a forensic pathologist with experience in forensic radiology. The radiological diagnosis was performed using the axial, coronal, and sagittal orientation slices. 3D reconstructions of the bone were also performed, for visualization purposes only. The autopsy of the body followed the same day after radiological examination.

### Anthropological analyses

Following the autopsy, the almost complete burnt cranium was sampled for anthropological analyses. For the cleaning process, the bone was put in a steam oven at 60°C temperature for ⁓2 days in an enzyme mixture for bone preparation containing 40 g of ENZYRIM, 40 g of soap, and 2 L of water. After cleaning, the bones dried at room temperature for approximately 3 more days. The dried fragments were then reconstructed using wood glue and investigated macroscopically. Each feature of the bone was scrutinized, any coloration or discoloration was noted, the length of each lesion was assessed using sliding callipers, and photo documentation was taken.

The best way to analyse fractures on charred bones is to understand the mechanism of heat-induced fractures (HIF). Four different kinds of bone damage were identified by Divya et al. [7], namely (a) antemortem or perimortem, traumatic bone fractures, (b) postmortem (prefire), nontraumatic bone fractures; (c) HI bone fractures, and (d) indirect HI bone fractures. However, due to bone alterations caused by the heat, classifications of fractures can be highly difficult and analyses can be hampered.

We assessed each fracture to differentiate between BFT and HIF. Plastic deformations correspond to irreversible deformations of the bone under the effect of a mechanical stress. If the stress is too great, the next stage is fracture [21, 22]. Deformation of the bone is indicative of a perimortem event when the bone was still in a “fresh” state [21].

In the case of HIF, fracture lines are often found within the carbonized regions [1, 23]. Bone is comprised in major parts of collagen and hydroxyapatite [24]. Whilst collagen provides tensile strength, the hydroxyapatite crystals sustain the hardness of the bone. When exposed to fire or to extreme heat, the elasticity of the bone decreases due to collagen dehydration. This leads to significant alterations of the bone structure and thus to shrinkage and deformation of the bone [25]. This modification may lead eventually to bone cracks, which can be found in dark brown, black, grey, or even white bone areas.

### Additional MDCT- and 3D surface scan

An MDCT-scan of the cranium (same MDCT Lightspeed VCT 64 as mentioned above) was performed before cleaning; the images were used as additional reference for the bone reconstruction. Two additional MDCT-scans after the cleaning process were conducted (one before and one after the reconstruction), as well as a 3D-surface scan of the reconstructed cranium. Different parameters for the MDCT scans were used for the three MDCT scans (Table 1).

**Table 1:** MDCT-scan parameters used for the three different scans using CT Lightspeed VCT 64, GE Healthcare.

	Scan type	kV	mA	Tube rotation (s)	Thickness (mm)	Slice interval (mm)	SFOV (cm)	Pitch	ASiR	Algorithm
Total Body MDCT-scan	Helical	120	200–300	1	1.250	1.0	50	1.375:1 (=2)	40	Standard
MDCT-scan of the skull before maceration	Helical	120	400	1	0.625	0.3	32	0.516:1 (=0.5)	–	Bone + reconstruction in Standard
MDCT-scan of the skull after maceration	Helical	120	120	1	0.625	0.3	32	0.516:1 (=0.5)	–	Bone + reconstruction in Standard (thickness 1.25 mm; slice interval 0.625 mm)

kV: kilovoltage; mA: milliampere; MDCT: multidetector computed tomography; SFOV: scan field of view; pitch: ratio of the table increments to the total nominal beam width for the MDCT-scan; ASiR: adaptive statistical iterative reconstruction.

3D reconstructions were conducted to better visualize the fractures on the skull. For the 3D surface scan of the reconstructed skull as well as for the assumed used object (in this case a hammer), a Gesellschaft für optische Messtechnik (GOM) ATOS compact scan 5 M (GOM Metrology GmbH, Braunschweig, Germany) was used, calibrating a measuring volume of 150 mm × 110 mm × 110 mm on a camera distance base of 300 mm [26].

## Results

### Radiological analysis of the MDCT scans before autopsy

**Figure 1:**
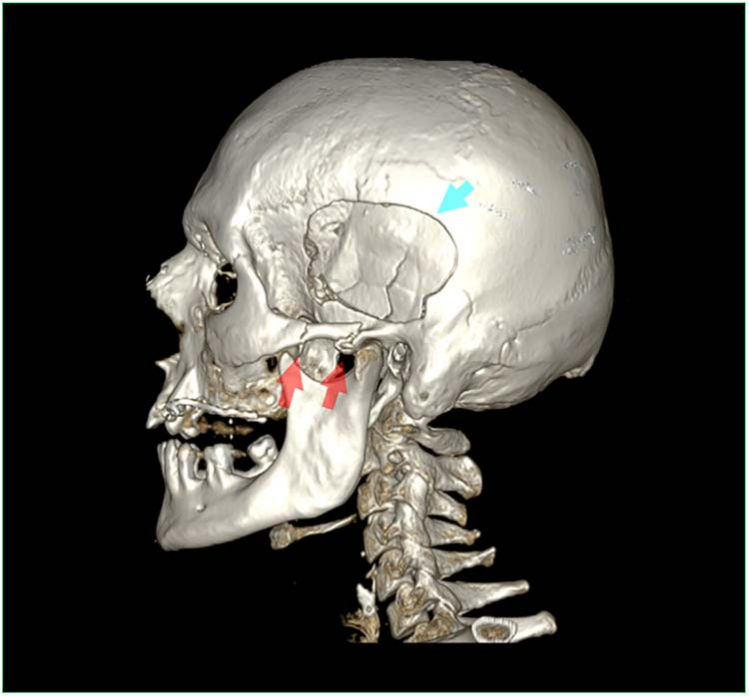
3D reconstruction of the skull and the cervical spine (multidetector computed tomography scan before autopsy), showing the left side of the skull and the cervical spine with visualization of the oval multifragmentary depressed skull fracture in the left temporo-parietal region (blue arrow) and the bifocal fracture of the left zygomatic bone (red arrows).

The images of the cranial MDCT-scan showed an irregularity of the skin layer of the face and an absence of skin and soft tissues regarding most of the skull and the nose. The main radiological diagnostic was an oval multifragmentary depressed skull fracture in the left temporo-parietal region, associated with a fracture of the left petrous bone (Figure 1). Moreover, the MDCT-scan analysis revealed fractures of the anterior and lateral wall of the left maxillary sinus and left zygomatic bone, and a small bone fragment next to the coronoid process of the left mandible as a left maxillary and bilateral sphenoidal hemosinus. Furthermore, a juxta-dural frontal bilateral hematoma, associated to small bone fragments, was visualized.

### Observation made on the skull during autopsy

The autopsy revealed several fractures and bruising on the skull. There was an oval depressed fracture visible on the left temporo-parietal region with radial fracture lines extending to the left petrous bone, which was in accordance to the radiological examinations. Additionally, a probable bruising on the bone noted in the centre of this lesion and a haemorrhagic bleeding of the left temporal muscle.

Fractures of the facial bones, specifically the left maxillary, zygomatic, and left orbital bone were visible, with subcutaneous haemorrhagic suffusions.

Few cerebral cortical petechiae were observed on the left temporo-lateral and fronto-lateral regions, with acute left temporo-lateral cerebral bruising. A hematosinus could be traced on the left maxillary bone and both sides of the sphenoid.

Furthermore, the frontal part of the skull showed strong bilateral epidural heat hematoma.

### Anthropological analysis of the reconstructed skull bone

After cleaning the cranium, the carbonized areas were clearly visible, showing black and brown coloration on the external surface of the bone. Most affected areas were on the left frontal, above the left orbit, and on the left parietal bone. In addition to this, there were brown coloured areas on the right frontal and parietal bone.

The cranial vault and the petrous pyramids were separated from the rest of the skull, following the autopsy. Lesions on the cranium could be grouped in four main areas.

#### Lesions within carbonized bone on the right and left lateral area

**Figure 2:**
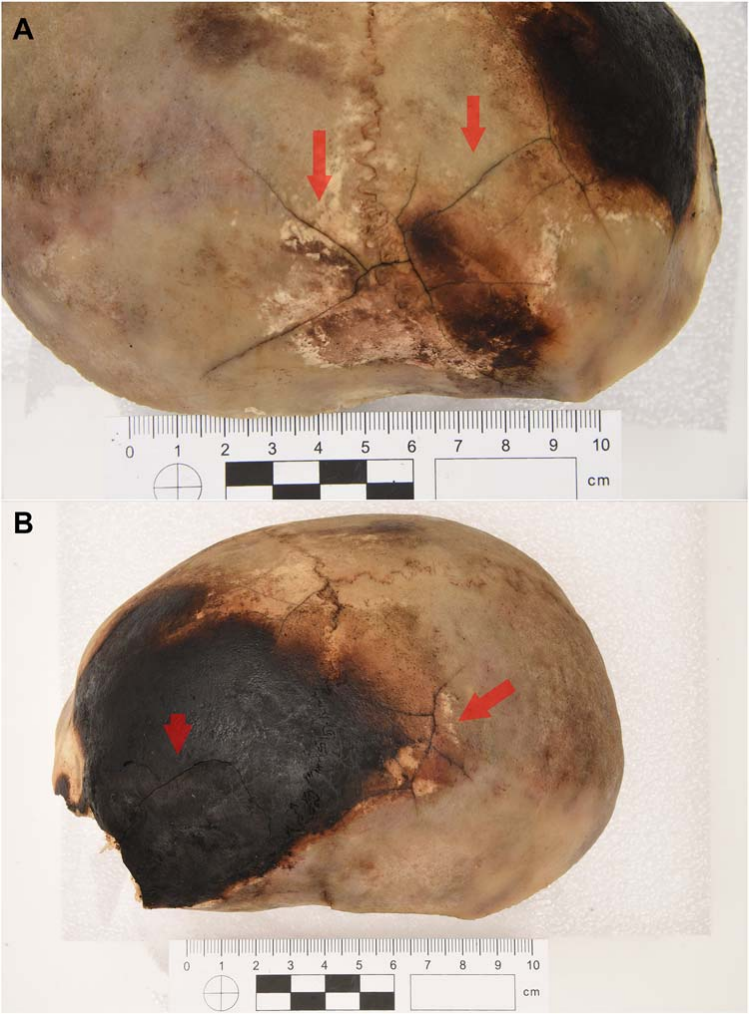
(A) The right side of the cranial vault from superior-lateral view. Fracture lines around the charred areas on the parietal and frontal bones are visible (red arrows). (B) The left side of the cranial vault from superior-lateral view. Several cracks, rather irregular, are visible on the lateral aspect of the left parietal bone and fracture lines on the left frontal bone within the dark charred parts (red arrows).

On the right lateral aspect of the skull, at the level of the parietal and frontal bone, there were fracture lines around the charred areas with dark brown colour change, visible as cracks (Figure 2A).

On the left lateral aspect of the skull, at the level of the parietal bone, several irregular fracture lines were visible. There were also cracks on the left charred frontal bone, in the central area, with colour changes into almost black colour (Figure 2B).

All described fracture lines were observed superficially on the outer table of the cranium, but not on the inner table.

#### A lesion above the left orbit

**Figure 3:**
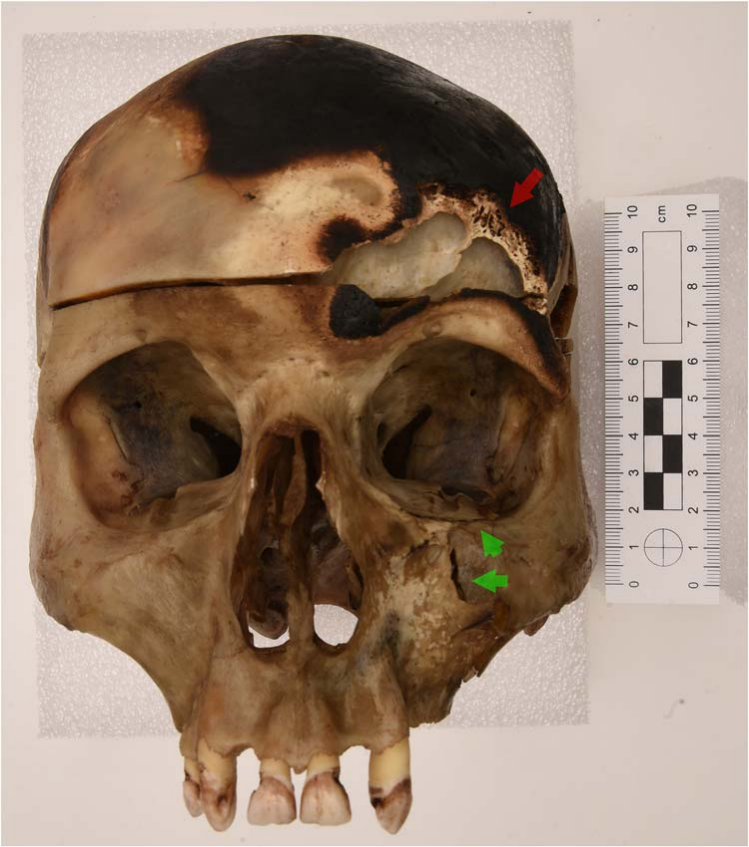
The skull in anterior view. There is a lesion on the left frontal bone above the left orbit. A lighter colour is visible on the upper edges of the lesion (red arrow). Furthermore, there is a lesion on the left side of the maxilla and the lower left orbital surface (green arrows).

There was a lesion on the left frontal bone, above the left orbit, including bone loss on the external aspect. The edges of this lesion partially showed a lighter, almost white colour, and the shape is somewhat oval but more irregular (Figure 3). The lesion measured ⁓2.1 cm × 4.6 cm. Charring with a black colour was visible around the lesion. Few bone fragments from this lesion were preserved as charred black bone splinters, which could not be reconstructed.

#### Lesions on the left temporal and parietal bones

**Figure 4:**
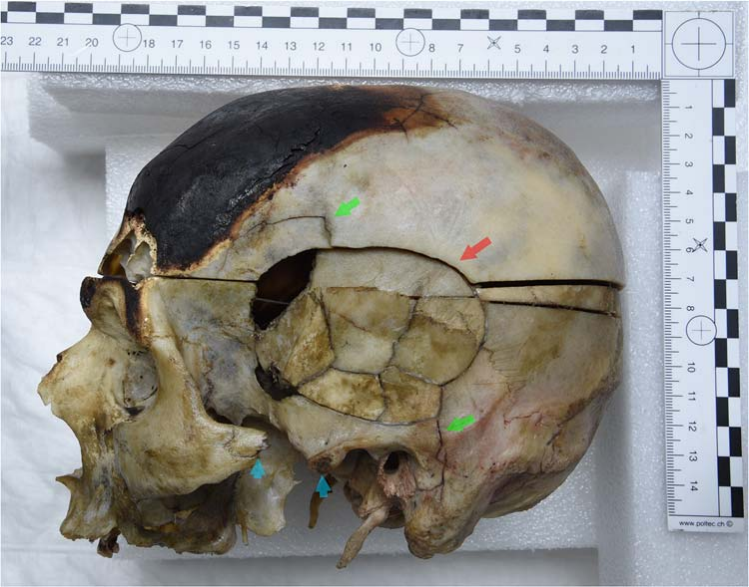
The skull after reconstruction in left lateral view. The lesion on the left temporal and parietal bones is visible. The lesions comprise several bone fragments and show plastic deformations. The lesion shows an oval/round shape with a concentric fracture line (red arrow) and radial line (green arrows). The left zygomatic process shows a complete breakage with a separated bony portion (blue arrows).

There was a lesion on the left lateral aspect of the skull, at the level of the left temporal and parietal bones. The edge of the temporal bone was separated from the parietal bone. The lesion showed an oval/round shape with a concentric fracture line, radial fissures, as well as a central area with seven bone fragments preserved and some bone splinters that were missing following preparation (Figure 4). The lesion measured a total of ⁓7 cm long along its horizontal axis, and 5 cm across its vertical axis. The temporal bone fragments are deformed, and dark staining was visible in the lesion centre after reconstruction.

#### A lesion on the left zygomatic and maxillary bone

The left zygomatic process showed complete breakage with a separated bony portion (Figure 4). Furthermore, there was a lesion on the left side of the maxilla and the lower left orbital surface (Figure 3).

None of the described lesions showed new bone formation macroscopically (e.g. bone callus).

## Discussion

The identification of perimortem trauma is one of the most challenging tasks for forensic anthropologists, and one that is even more complicated when looking at burnt human remains. As described by Kemp [27] anthropologists have to distinguish postmortem changes from trauma but also consider the fact that these changes may also mask actual trauma. In addition to this, the exposure of the bone to extensive heat lead to changes of the morphology and thus may lead to misleading evidence. However, in some instances traumatic events, especially on the skull, can still be identified after heat exposer as shown by e.g. Franceschetti et al. [28] with their analyses on cremated remains.

Anthropological analyses of the skull in the presented case were consistent with the radiological and autopsy report. It was possible to reconstruct the various lesions on the dry bone. Four main areas of lesions on the cranium could be identified and differentiated into three main types of fractures (based on the description in Divya et al. [7]): HIF, indirect heat-induced fracture (iHIF) and BFT.

### Heat induced fractures

Several fracture lines were noted, located roughly in the centre of the discoloration and carbonized portion of the skull, mainly on the left and right lateral areas. As soft tissues were mainly burned in this area, no further investigations on bruises and abrasions as deeper wounds could be made. The fracture lines were visible on the outer table, but not on the inner table. This was confirmed by the radiological analyses showing only slight superficial lines on the outer table of the cranium. Furthermore, no plastic deformation on these fractured areas were visible. Although a blunt force causing the fracture lines could not be completely excluded, analyses suggest a HIF lines as the most likely explanation.

### Indirect heat induced fractures

**Figure 5:**
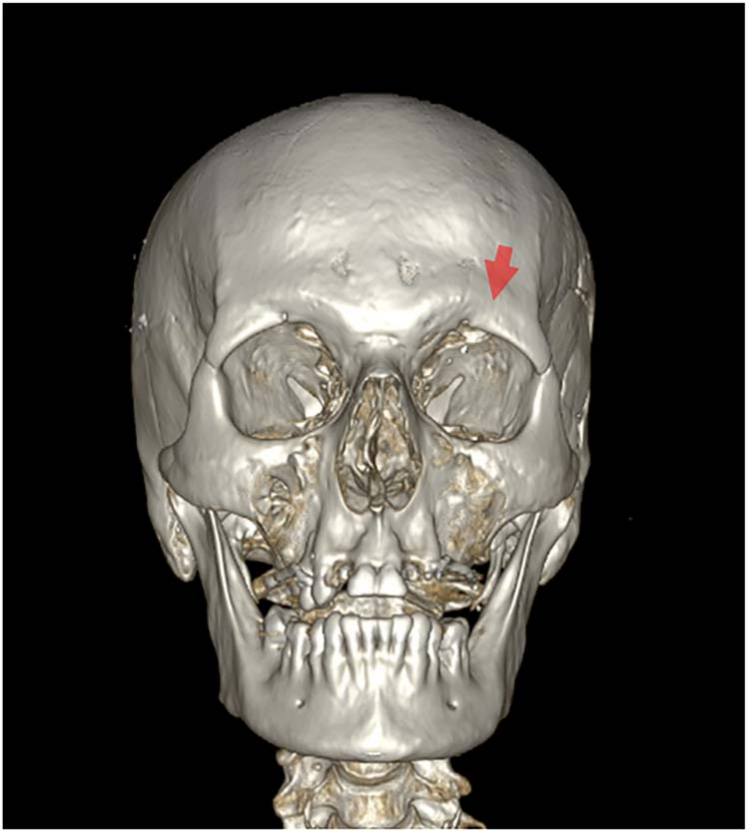
3D reconstruction of the skull (multidetector computed tomography scan before autopsy), showing the front side of the skull before autopsy. No fracture is visible on the left frontal bone, above the left orbit (red arrow).

In addition to the described fracture lines, there was a fracture presenting as a fragmented bone area on the left frontal above the left orbit. The lesion showed partially lighter coloured margins, indicative of a postmortem fracture [29]. The MDCT-scan performed prior to autopsy showed no fragmentation in this region (Figure 5). *Postmortem* damage is the most likely explanation based on the anthropological and radiological analyses. Furthermore, the autopsy revealed strong bilateral epidural heat hematoma in the frontal part, which confirms the anthropological results. The lesion was situated within the highly calcified bone area. The heat might have caused micro fractures and cracks, which were not visible on the MDCT-scan. The outer table of the left frontal bone might have burst open as a consequence, probably during autopsy, which then eventually lead to the fracture of the bone and loss of calcified bone splinters during the cleaning process. This lesion could be classified as indirect HIF. As described by Divya et al. [7], “After thermal exposure, the bone can be subjected to forces or influences, which can lead to post-fire fractures. These fractures are not directly caused by exposure to heat but indirectly the result of structural and molecular HI-changes of the bone matrix, in combination with forces or influences acting on the bone”.

### Blunt force trauma

The MDCT-scan revealed an oval multifragmentary depressed skull fracture on the left temporo-parietal region, associated with a fracture of the left petrous bone and factures of the anterior and lateral wall of the left maxillary sinus and the left zygomatic bone. Autopsy confirmed the depressed fracture with multiple fragments on the left side visualized during the MDCT-scan analysis.

Anthropological analyses are congruent with the radiological and autopsy results. After manual reconstruction of the fragmented area, plastic deformations of the fragments were clearly visible. In addition to this, we noted that the central zone, which corresponds to a probable impact zone, was associated with an oval shaped continuity solution. The described features are indicative of a blunt mechanism, such as a blow, a fall, or item(s) dropped on a person [22, 30]. Furthermore, there were also radial fracture lines and a concentric fracture within this central zone (Figure 4). After the manual reconstruction, a circular area was visible, with a darker colour, which could be due to bone bruising. All the characteristics described indicate a trauma, which may have occurred perimortem [21, 30].

**Figure 6:**
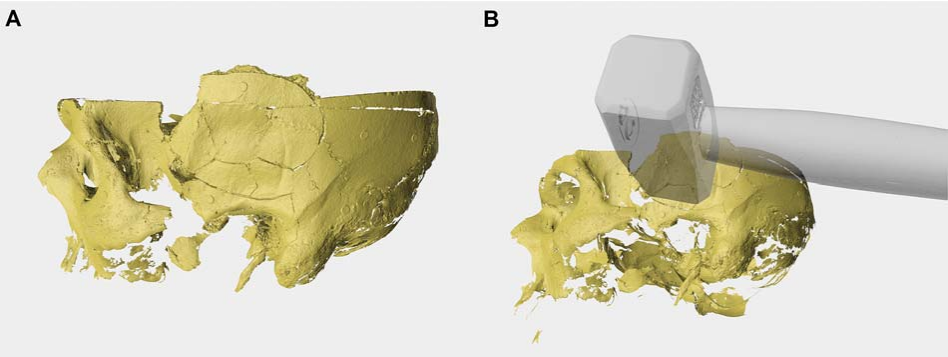
(A) 3D-model of the surface scanned reconstructed skull (right lateral view). (B) Virtual repositioned hammer on the fractured skull. For better visualization, the opacity of the hammer has been reduced.

The reconstructed lesion was 3D surface scanned for additional superimposition with the alleged object used by the perpetrator. Although the exact sequence of events were not known, the perpetrator was presumably right handed and could have hit the victim standing in front of him. This, however, cannot be stated with certainty, as the victim could have been lying down. The hammer found in the bag was scanned and virtually repositioned, true to scale, over the reconstructed fractured area. The results showed that a blunt force mechanism using the hammer was possible (Figure 6). However, as the superimposition was made only with the hammer, other possible objects cannot be excluded.

Furthermore, bone lesions were observed on the left zygomatic and left maxillary bone. The zygomatic and left maxillary lesions do not have all the elements required to classify them as a typical Le Fort fracture or a complete (tripod) fracture of the zygomatic bone [31, 32]. The fracture visible on the zygomatic arch, however, presents features that could be indicative of a blunt mechanism [30]. The left maxillary fracture may be part of an indirect mechanism, related to the consequence of the zygomatic arch fracture, or may be related to another direct trauma, independent of the one that generated the zygomatic arch fracture. Nevertheless, the analyses do not allow us to affirm one of these hypotheses with certainty. Although it is difficult to distinguish whether this lesion has occurred perimortem or postmortem, an HI postmortem event is less likely because of the fracture morphology [21, 30].

It was not possible to estimate a timeline for these traumatic injuries. Depending on the object that may have caused the trauma, including the suspected hammer, it is possible that the entire fractured area was affected by a single blow. However, it is not possible to exclude that the lesions on the left side of the neuro- and viscerocranium were the results of multiple impacts.

## Conclusions

This case offered an opportunity to apply an interdisciplinary approach to the analysis of burnt human remains, which are one of the most challenging cases in forensic anthropology. Anthropological findings were consistent with those made through MDCT-scan analysis and autopsy. The morphology of the lesions is compatible with at least one impact on the left side of the cranium, but multiple impacts cannot be ruled out.

With the help of anthropological analyses in a multidisciplinary approach, it was possible to analyse and differentiate the multiple lesions on the heavily burnt skull. In conclusion, this case illustrates the necessity of addressing instances where bodies are taphonomically altered in an interdisciplinary manner.

Limitations to this process includes loss of fragile bone fragments during the sampling/cleaning procedure. Furthermore, when force is applied to a bone, plastic deformation occurs. The deformation, however, does not disappear with the fracture. It remains and makes fracture reconstruction difficult as fragments held in shape by soft tissue might overlap or, in opposition, there might be gaps in between fragments. MDCT-scans of the body taken prior to autopsy and sampling can guide the anthropologists in their reconstruction. Finally, the line between “perimortem” and “postmortem” trauma is difficult to apprehend in forensic anthropology. Indeed, a bone will retain its potential to break in a way that could be interpreted as “perimortem” as long as it is “fresh”, i.e. as long as it has not lost its tensile properties. Since taphonomic variables that might slow or accelerate this process are numerous and each have their own properties (heat, explosions, exposition to hot or cold weather, dry or humid environments, etc.), forensic anthropologists have to contemplate all eventualities before making their conclusions.
